# Prevalence of major risk factors for tuberculosis in COVID-19 active case-finding camps in India

**DOI:** 10.5588/pha.26.0025

**Published:** 2026-08-24

**Authors:** S. Arunachalam, T. Chandra, A. Basu, M. Gandhi, S. Shrivastava, A. Shekhawat, M. Singh, A. Khanna, S. Mannan

**Affiliations:** William J. Clinton Foundation, New Delhi, India.

**Keywords:** tuberculosis, undernutrition, diabetes mellitus, tobacco use, alcohol consumption, digital chest radiography

## Abstract

**BACKGROUND:**

TB) remains a major public health challenge in India, driven by undernutrition, diabetes, alcohol use, and tobacco exposure. Under the Global Fund–supported COVID-19 Response Mechanism (C19RM), we implemented integrated active case finding (ACF) and risk screening to estimate the prevalence of key risk factors among individuals with TB. We assessed their combined influence on detection yield, and examined state-wise variation in TB and non-communicable disease (NCD) comorbidity patterns.

**METHODS:**

This cross-sectional analysis included 17,288 C19RM-supported ACF camps across 11 states. Screening combined digital chest X-ray, molecular diagnostics, and NCD risk assessment, including anthropometry, self-reported diabetes and hypertension, opportunistic blood sugar testing, and self-reported tobacco and alcohol use. Multivariable logistic regression estimated adjusted odds ratios (aORs) for TB diagnoses.

**RESULTS:**

Among 1,383,058 individuals screened, 11,012 TB cases were identified (0.8% yield). Undernutrition (BMI <18.5 kg/m^2^) accounted for 40% of cases and showed the strongest association (aOR 3.89). Alcohol (aOR 1.62) and tobacco use (aOR 1.51) were independently associated with TB, while diabetes showed an inverse adjusted association despite 10% prevalence. Interaction analyses indicated higher TB odds with coexisting risk factors, with geographic clustering observed.

**CONCLUSION:**

Integrated TB–NCD screening revealed clustering of nutritional, behavioural, and metabolic risks, supporting risk-prioritised ACF strategies.

Tuberculosis (TB) remains a major public health challenge in India, which accounts for nearly one quarter of the global TB burden.^1^ Despite progress under the National TB Elimination Programme, India continues to report the highest absolute number of TB cases globally.^1^ The WHO End TB Strategy emphasises early diagnosis, systematic screening of high-risk populations, and integrated management of comorbidities to reduce TB incidence and mortality.^1^ Non-communicable diseases (NCDs) and behavioural risk factors are increasingly recognised as critical determinants of TB risk, disease severity, transmission dynamics, and treatment outcomes.^2,3^ Diabetes mellitus increases the risk of active TB by two to three fold and is associated with delayed sputum conversion and poor outcomes.^4,5^ Undernutrition is the single largest population-attributable risk factor for TB in India, contributing substantially to incident disease and mortality.^6^ Alcohol consumption and tobacco use further increase TB susceptibility through immune dysregulation, increased exposure risk, delayed care-seeking, and treatment non-adherence.^7,8^

The COVID-19 pandemic disrupted routine TB services, leading to declines in case notifications globally.^1^ In response, The Global Fund–supported COVID-19 Response Mechanism (C19RM) enabled large-scale TB active case finding (ACF) integrated with NCD screening across high-burden settings in India. These camps deployed digital chest X-ray (CXR), computer-aided detection (CAD), and rapid molecular diagnostics to identify undiagnosed TB, particularly among vulnerable populations. However, there remains limited evidence describing the prevalence, interaction, and geographic distribution of NCD risk factors among TB patients identified through integrated ACF platforms at scale.

Our study is an attempt to better understand these patterns to optimise risk-stratified screening and integrated TB and NCD care. Our primary objective was to estimate the prevalence of major NCDs and risk conditions among persons diagnosed with TB identified through C19RM-supported ACF camps. Secondary objectives were to assess the contribution of individual and interacting risk factors to TB detection yield, and examine state-wise variation in NCD–TB comorbidity patterns.

## METHODS

This cross-sectional descriptive and analytical study used routinely collected programmatic data from 17,288 C19RM-supported TB ACF camps conducted across 10 states/UTs in India between April 2023 and October 2025.

### Screening and diagnostic procedures

All beneficiaries underwent: symptom screening; digital CXR with CAD support; sputum examination using rapid molecular diagnostics; and NCD risk-factor assessment including body mass index (BMI) measurement, self-reported diabetes, random blood sugar (RBS), blood pressure, tobacco use, and alcohol use. TB diagnoses were classified as microbiologically confirmed TB (MBC), or clinically diagnosed TB.

### Variable definitions and measurement procedures

Anthropometric measurements were obtained during camp registration using locally available weighing scales and stadiometers or height measurement scales. Measurements were performed by trained ASHA workers or camp staff following routine operational procedures. BMI was calculated as weight (kg)/height (m^2^), and undernutrition was defined as BMI <18.5 kg/m^2^ according to WHO classification. Blood pressure screening was performed using available digital or manual sphygmomanometers under routine field conditions; however, device standardisation and repeated measurements could not be uniformly ensured across all sites. Elevated blood pressure was defined operationally based on the screening threshold used in the respective state programme. Diabetes status was primarily based on self-report during verbal screening. RBS testing was performed opportunistically depending on local availability of consumables and staff capacity. RBS measurements were intended for presumptive screening rather than definitive diagnosis. Individuals with RBS >200 mg/dL were referred for confirmatory evaluation at nearby health facilities. Tobacco and alcohol use were self-reported and recorded during standardised verbal risk assessment interviews conducted by camp staff. The details were finally recorded in the Radiological Information System (RIS) developed by the C19 team for maintaining a detailed record of beneficiaries.

### Statistical analysis

Multivariable logistic regression models were constructed to estimate adjusted odds ratios (aORs) and 95% confidence intervals (CIs) for 1) MBC TB and 2) all TB diagnoses (MBC + clinically diagnosed). Covariates entered into the models included age, sex, BMI category, self-reported diabetes, elevated RBS status, elevated blood pressure status, tobacco use, alcohol use, and tribal status where available. Interaction terms were prespecified based on biological plausibility and prior literature: low BMI × diabetes; low BMI × alcohol use; diabetes × tobacco use.

Model outputs were interpreted as associative rather than causal due to the cross-sectional nature of the data. Missing data were handled using complete-case analysis. Analyses were conducted using de-identified individual-level programmatic data extracted from the centralised RIS.

### Ethical statement

Formal ethics committee approval was not required for this study as it involved the retrospective analysis of de-identified, routine programmatic data. The data were collected as part of standard public health surveillance and ACF activities. All personal identifiers were removed prior to analysis to ensure patient confidentiality; therefore, the requirement for informed consent was waived in accordance with national guidelines for the use of secondary programmatic data for public health evaluation.

## RESULTS

A total of 1,383,058 beneficiaries were screened; 11,012 individuals were diagnosed with TB (overall yield: 0.8%). The mean age of TB patients was 49.1 ± 18 years. Men comprised 62% of TB cases despite representing only 43% of those screened (see Figure).

**FIGURE.**
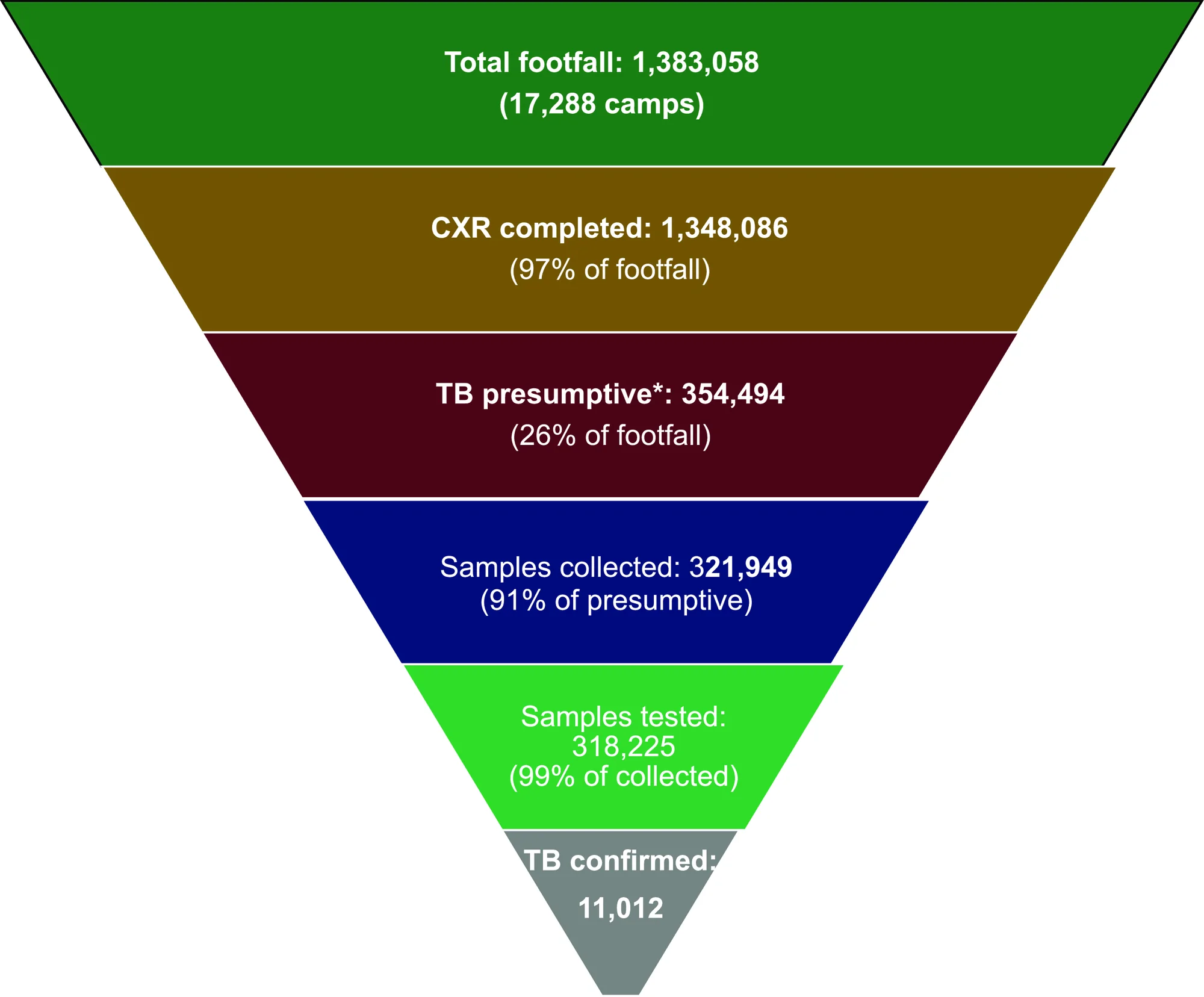
Bird’s eye view of the tuberculosis (TB) active case-finding study supported by the COVID-19 Response MechanismC19RM project. *TB presumptive based on symptom and/or AI suggestive or medical officer. CXR = chest X-ray.

Undernutrition (BMI <18.5 kg/m^2^) was present in 16% of screened beneficiaries but accounted for 40% of TB diagnoses. Tobacco use (34%) and alcohol use (15%) were common among TB patients, while self-reported diabetes was reported by 10% (Table 1).

**TABLE 1.** Characteristics of individuals screened for TB and diagnosed with TB.

Characteristic	Total screened (n = 13,83,058)	TB confirmed (n = 11,012)	Non-TB (n = 13,82,046)
Age, mean ± SD	46.4 ± 17	49.1 ± 18	46.4 ± 17
Male sex, n (%)	595,437 (42.9%)	6,791 (61.7%)	588,646 (42.7%)
Tribal population, n (%)	40,572 (2.9%)	432 (3.9%)	40,140 (2.9%)
BMI <18.5 kg/m^2^, n (%)	218,123 (15.7%)	4,446 (40.4%)	213,677 (15.5%)
Self-reported diabetes^A^ n (%)	179,616 (12.9%)	1,061 (9.6%)	178,555 (13%)
Elevated RBS (>140 mg/dL)^B^ n (%)	214,710 (15%)	1,478 (13%)	213,232 (15%)
Elevated BP (presumptive hypertension)^C^ n (%)	162,684 (11.7%)	1,339 (12.2%)	161,345 (11.7%)
Tobacco use, n (%)	373,071 (26.9%)	3,727 (33.8%)	369,344 (26.8%)
Alcohol use, n (%)	139,933 (10.1%)	1,620 (14.7%)	138,313 (10%)

TB = tuberculosis; BMI = body mass index; BP = blood pressure; RBS = random blood sugar.

A Self-reported diabetes refers to participant-reported prior diagnosis.

B Elevated RBS represents opportunistic screening values and not confirmed diabetes diagnosis.

C Blood pressure measurements were conducted under routine field conditions without centralised calibration verification.

### Individual risk factors and TB risk

Low BMI demonstrated the strongest statistical association with TB diagnosis (MBC aOR 3.89, 95% CI: 3.71–4.07; all TB aOR 3.69, 95% CI: 3.55–3.83). Alcohol (MBC aOR 1.62) and tobacco use (MBC aOR 1.51) were independently associated with increased TB risk. Self-reported diabetes showed an inverse association (MBC aOR 0.78), which should be interpreted cautiously given reliance on self-reported diabetes status, potential underdiagnosis, survivor bias, reverse causality, and incomplete biochemical screening (Table 2). RBS-based sensitivity analysis was also conducted but was underpowered due to low event counts and did not provide any significant result.

**TABLE 2.** Adjusted odds ratio (aOR) for important risk factors for TB (n = 11,012). Models adjusted for age, sex, tribal status, BMI category, diabetes status, tobacco use, alcohol use, elevated blood pressure, and elevated RBS where available.

Risk factor	Proportion among TB (%)	MBC aOR (95% CI)	All TB aOR (95% CI)
Low BMI	40	3.89 (3.71–4.07)	3.69 (3.55–3.83)
Alcohol use	15	1.62 (1.52–1.73)	1.54 (1.46–1.63)
Tobacco use	34	1.51 (1.44–1.58)	1.40 (1.34–1.45)
Self-reported Diabetes	10	0.78 (0.72–0.84)	0.72 (0.67–0.76)

TB = tuberculosis; BMI = body mass index; RBS = random blood sugar; MBC = microbiologically confirmed; CI = confidence interval.

### State-wise distribution of risk factors

Marked inter-state variation was observed in the distribution of key risk factors among total TB patients. The proportion of TB patients with low BMI was highest in Rajasthan and Uttarakhand and lowest in Meghalaya and Ladakh. Alcohol use among TB patients was most prevalent in Kerala and Tamil Nadu. Similarly, tobacco use was highest in Kerala and Uttarakhand. In contrast, diabetes prevalence among TB patients was highest in Kerala and Tamil Nadu. Overall, southern states demonstrated higher metabolic and behavioural risk-factor burden, whereas northeastern and high-altitude regions showed consistently lower proportions across most risk factors (Table 3).

**TABLE 3.** States with highest and lowest proportion of important NCDs among people with TB.

Risk factor	Highest state (%)	2nd highest state (%)	Lowest state (%)	2nd lowest state (%)
Low BMI	Rajasthan (58.5%)	Uttarakhand (44.6%)	Meghalaya (12.5%)	Ladakh (15.1%)
Alcohol	Kerala (41.5%)	Tamil Nadu (27.7%)	Ladakh (0)	Haryana (8.22%)
Tobacco	Kerala (58.5%)	Uttarakhand (41.9%)	Ladakh (0)	Meghalaya (12.5%)
Diabetes	Kerala (36.6%)	Tamil Nadu (25.9%)	Meghalaya (0)	Ladakh (6.6%)

TB = tuberculosis; BMI = body mass index; NCDs = non-communicable diseases.

### Interaction effects

Statistical interaction analyses suggested that concurrent exposure to nutritional, metabolic, and behavioural risk factors was associated with higher odds of TB than expected from individual effects alone. The coexistence of low BMI and diabetes was associated with 30% higher odds of MBC TB and 35% higher odds of all TB compared with the expected effect of each factor alone. Similarly, low BMI combined with alcohol use showed a modest but significant synergistic effect, increasing the odds of MBC TB by 22% and all TB by 16%. In addition, a strong interaction was noted between diabetes and tobacco use, with a 35% increase in odds of MBC TB and a 24% increase in odds of all TB, indicating amplified TB risk in individuals with concurrent metabolic and behavioural vulnerabilities (Table 4). No significant interaction was observed between low BMI and tobacco. However, these findings should be interpreted cautiously, as interaction estimates derived from cross-sectional observational data do not establish biological synergy or causal interaction.

**TABLE 4.** Adjusted odds ratios for interaction terms associated with TB (n = 11,012).

Interaction	MBC aOR (95% CI)	All TB aOR (95% CI)
Low BMI × diabetes	1.30 (1.10–1.54)	1.35 (1.17–1.56)
Low BMI × alcohol	1.22 (1.07–1.39)	1.16 (1.04–1.29)
Diabetes × tobacco	1.35 (1.15–1.58)	1.24 (1.08–1.41)

TB = tuberculosis; MBC = microbiologically confirmed; OR = odds ratio; CI = confidence interval.

## DISCUSSION

This large multi-state analysis demonstrates that undernutrition is the dominant driver of TB risk among individuals identified through integrated ACF. These findings are consistent with prior Indian modelling studies estimating that undernutrition accounts for a substantial proportion of incident TB.^6^ Malnutrition impairs cell-mediated immunity and macrophage function, increasing susceptibility to *Mycobacterium tuberculosis* infection and disease progression.^3^ The strong association between low BMI and TB should also be interpreted in the context of potential reverse causality, since active TB itself commonly results in weight loss and cachexia. Consequently, low BMI observed at screening may represent both a predisposing factor and a consequence of active disease.

Alcohol and tobacco use were independently associated with increased TB risk, particularly MBC disease. Meta-analyses demonstrate that alcohol use increases TB incidence and mortality,^7^ while tobacco exposure increases TB infection, disease progression, and poor treatment outcomes.^8^

The inverse association observed with self-reported diabetes likely reflects substantial measurement and ascertainment limitations rather than a protective effect. Diabetes status relied primarily on participant self-report, which may underestimate true prevalence in underserved populations with limited prior access to diagnostic services. Additionally, individuals with advanced TB disease may experience transient reductions in blood glucose or weight-related metabolic alterations that complicate diabetes ascertainment at the time of screening. Evidence from TB–diabetes literature suggests that biochemically confirmed diabetes or chronic hyperglycaemia is more strongly associated with TB risk than self-reported measures or single-point glucose testing.^9^ Prospective cohort studies using biochemical screening consistently show increased TB risk among individuals with diabetes.^4,5^ Selection bias related to incomplete RBS testing and referral-based confirmation may have further influenced observed associations as individuals undergoing biochemical testing may not be representative of the broader screened population.^10^

The observed geographic variation in risk-factor distribution aligns with known epidemiological patterns in India. Higher proportions of diabetes and alcohol use among TB patients in southern states such as Kerala and Tamil Nadu are consistent with regional NCD burdens reported in national surveys.^11,12^ Conversely, lower prevalence in northeastern and high-altitude regions reflects differing lifestyle and metabolic profiles.^13^ The high burden of undernutrition in northern states parallels evidence identifying low BMI as a key driver of TB in India.^6^ These findings reinforce the need for region-specific, integrated TB–NCD strategies, as recommended in recent programmatic and epidemiological studies.^14^

The interaction analyses suggest clustering of multiple vulnerabilities within high-risk populations wherein undernutrition, metabolic disease, and substance use interact synergistically to amplify TB risk.^15,16^ Identification of such high-risk phenotypes can enhance efficiency of ACF programmes.

Strengths of this study include its large scale, multi-state implementation across 11 states and union territories, and the integration of concurrent NCD risk-factor screening within a real-world ACF platform, providing one of the largest programmatic datasets on NCD–TB comorbidity patterns in India. Several limitations should be considered when interpreting these findings. First, the cross-sectional design precludes causal inference; observed associations between NCD risk factors and TB cannot establish temporal directionality. Second, reliance on self-reported data for diabetes, tobacco, and alcohol consumption likely underestimates true prevalence in this low-literacy population, potentially biasing observed odds ratios toward the null. Third, RBS testing lacked fasting or postprandial distinction; furthermore, the RBS-based sensitivity analysis was underpowered due to the low proportion of participants meeting the diagnostic threshold of >200 mg/dL. Fourth, while data quality was monitored via a centralised RIS, the use of programmatic data across 11 states introduces inherent variability in measurement standardisation. Fifth, self-selection bias among ACF camp attendees limits generalisability, as participants may systematically differ from the broader community in health-seeking behaviour. Sixth, substantial inter-site heterogeneity likely existed in anthropometric measurements, blood pressure recording practices, and RBS testing procedures due to the operational nature of the camps. Calibration procedures, duplicate measurements, fasting status, and standardised quality-control procedures could not be uniformly verified across all participating states. Seventh, alcohol and tobacco exposure were assessed as binary self-reported variables without quantification of duration or intensity, limiting dose–response interpretation. Finally, because camp attendance was voluntary, the screened population may not represent the underlying community prevalence of TB or NCD risk factors.

## CONCLUSION

Integrated TB–NCD screening through ACF platforms reveals substantial clustering and interaction of nutritional, behavioural, and metabolic risk factors among TB patients. Risk-stratified ACF prioritising undernourished individuals, substance users, and those with combined vulnerabilities may significantly enhance bacteriological TB detection in high-burden settings.
